# 1326. Mupirocin Resistance in *Staphylococcus aureus* in Pediatric Patients for Ten Years

**DOI:** 10.1093/ofid/ofac492.1156

**Published:** 2022-12-15

**Authors:** Hwanhee Park, Doo Ri Kim, Kyung-Ran Kim, Jong Min Kim, Hee Jae Huh, Nam Yong Lee, Yae-Jean Kim

**Affiliations:** Soonchunhyang University Bucheon Hospital, Bucheon, Kyonggi-do, Republic of Korea; Samsung medical center, Seoul, Seoul-t'ukpyolsi, Republic of Korea; Gyeongsang National University Changwon hospital, Changwon, Kyongsang-namdo, Republic of Korea; Myungji Hospital, Goyang, Kyonggi-do, Republic of Korea; Samsung medical center, Seoul, Seoul-t'ukpyolsi, Republic of Korea; Samsung Medical Center, Seoul, Seoul-t'ukpyolsi, Republic of Korea; Samsung Medical Center, Seoul, Korea, Seoul, Seoul-t'ukpyolsi, Republic of Korea

## Abstract

**Background:**

Mupirocin has been recommended for decolonization in methicillin-resistant *Staphylococcus aureus* (MRSA) carriers for recurrent skin and soft tissue infections and treatment for impetigo. However, the indiscriminate use of mupirocin causes mupirocin resistance, associated with decolonization failure. The aim of the study was to investigate the epidemiology of mupirocin resistance and to identify clinical characteristics among children in a single center.

**Methods:**

From January 2011 to October 2020, we retrospectively analyzed the epidemiology of antibiotic resistance and clinical characteristics of pediatric patients under 19 years old in whom *S. aureus* was firstly isolated at any body site.

**Results:**

Of the 3,414 *S. aureus* isolates, 46% (1569/3414) were methicillin-resistant, and 20.3% (692/3414) were mupirocin resistant. Among MRSA, Mupirocin-resistant (MupR) was 22.6% (354/1569), and among methicillin-sensitive S. aureus (MSSA), MupR was 18.3% (338/1845) (*P*< 0.001). The median age of MupR MRSA patients was 0.14 years (interquartile range 0.04-0.79), and the median age of MupR MSSA patients was 5.0 years (interquartile range 2.0-8.1) (*P*=0.000). Of 692 MupR *S. aureus*, 94.2% (652/692) were mainly detected in the skin. MupR MRSA was most frequently isolated in the neonatal intensive care unit (40.1%, 142/354), but MupR MSSA was most frequently isolated in the outpatient setting (81.4%, 275/338) (*P*< 0.001). Of these, 43% (119/275) patients were diagnosed with atopic dermatitis. By age, mupR MRSA was the more commonly isolated in infants younger than one year (77.4%, 274/354), and MupR MSSA was more commonly isolated in children older than three years old (65.4%, 221/338) (*P*< 0.001). The frequency of MupR MRSA and MSSA showed an increase in trend over time (*P*< 0.001). Among other topical agents, 6.5% (102/1569) of MRSA was resistant to fusidic acid.

**Conclusion:**

As mupirocin resistance gradually increases, a test for mupirocin susceptibility should be performed before applying the skin lesions or decolonization for MRSA. In addition, clinicians should carefully prescribe mupirocin to prevent the development of MupR *S. aureus.*

**Disclosures:**

**Yae-Jean Kim, MD, PhD**, Janssen: Grant/Research Support|Korean Society of Pediatric Infectious Diseases: Grant/Research Support|Ministry of Trade, Industry and Energy: Grant/Research Support|MSD: Grant/Research Support.

